# VGLUTs in Peripheral Neurons and the Spinal Cord: Time for a Review

**DOI:** 10.1155/2013/829753

**Published:** 2013-11-20

**Authors:** Pablo R. Brumovsky

**Affiliations:** Faculty of Biomedical Sciences, Austral University, Avenida Juan D. Perón 1500, 1629AHJ Pilar, Buenos Aires, Argentina

## Abstract

Vesicular glutamate transporters (VGLUTs) are key molecules for the incorporation of glutamate in synaptic vesicles across the nervous system, and since their discovery in the early 1990s, research on these transporters has been intense and productive. This review will focus on several aspects of VGLUTs research on neurons in the periphery and the spinal cord. Firstly, it will begin with a historical account on the evolution of the morphological analysis of glutamatergic systems and the pivotal role played by the discovery of VGLUTs. Secondly, and in order to provide an appropriate framework, there will be a synthetic description of the neuroanatomy and neurochemistry of peripheral neurons and the spinal cord. This will be followed by a succinct description of the current knowledge on the expression of VGLUTs in peripheral sensory and autonomic neurons and neurons in the spinal cord. Finally, this review will address the modulation of VGLUTs expression after nerve and tissue insult, their physiological relevance in relation to sensation, pain, and neuroprotection, and their potential pharmacological usefulness.

## 1. How VGLUTs Became the “Gold Standard” for the Identification of Glutamatergic Neurons

Before focusing on the presence and role of vesicular glutamate transporters in neurons in the periphery and the spinal cord, it is important to begin with some historical facts on how it was that glutamatergic neurons were identified in the nervous system. Several decades of research established that glutamate is the major excitatory neurotransmitter in the mammalian central nervous system (CNS) [1] and PNS, including dorsal root ganglion (DRG) and spinal cord neurons [2, 3]. However, the morphological phenotyping of glutamatergic neurons as well as glial cells was not to be a trivial matter.

First in accomplishing a major breakthrough were Storm-Mathisen, Ottersen, and their colleagues who, by means of careful electron microscopy methodologies and meticulous analysis, demonstrated glutamate-like immunoreactivity (Li) in several areas of the mouse, rat, guinea pig, and monkey brain and, importantly, its association to synapses [4–6]. This pioneering work led to the distinction of a “transmitter pool” in glutamatergic terminals, a “metabolic pool” in nonglutamatergic neurons, and a “glial pool” [7–9]. It also prompted the immunohistochemical analysis in sensory neurons, using antibodies against glutamate [10–14]. Subsequent methods to identify glutamatergic neurons were based on the immunohistochemical detection of enzymes like glutaminase, involved in the synthesis of glutamate [15, 16]. This was being reliably done for other neurotransmitters such as catecholamines, serotonin, acetylcholine, and also GABA [17]. More indirect approaches, like the identification of excitatory amino acid transporters (EAATs) located at the cell membrane, both of neurons and astrocytes, and critical for the removal of glutamate released at the synaptic cleft, also emerged [18, 19]. However, since glutamate is a major participant in cell metabolism, even for the synthesis of the inhibitory neurotransmitter GABA [7, 20], and not always the visualization of associated molecules guarantees the glutamatergic nature of neurons, an ideal marker was still sought.

A second breakthrough took place in the mid 1990s, when Ni and collaborators [21] revealed the presence of a brain-specific Na^+^-dependent inorganic phosphate transporter in the brain of rat pups. Further research showed that this transporter was specific to synaptic vesicles, acted as a vesicular glutamate transporter (VGLUT), and hence was termed VGLUT_1_ [22, 23]. Soon afterwards, VGLUT_2_ [24–30] and VGLUT_3_ [31–33] were discovered and characterized. Importantly, transfection of GABAergic neurons with DNA encoding VGLUT_1_ [23] or VGLUT_2_ [29] conferred the capacity to release both glutamate and GABA, confirming their role in glutamate loading of synaptic vesicles.

Thus, it was that the discovery of VGLUTs and the development of selective antibodies and *in situ* hybridization probes for their detection became the “gold standard” for the characterization of glutamatergic neurons in the brain and brainstem [34–37], the spinal cord [38–43], the peripheral nervous system (PNS) [30, 34, 36, 38, 44–66], and even glia [67–70]. It should, however, be mentioned that neurons coexpressing VGLUT_1_ [71] or VGLUT_3_ [31] and the GABAergic marker glutamate decarboxylase have been identified in developing rat hippocampal granule cells (GC), in adult rat hippocampal GCs under intense ionotropic or trophic factor stimulation [71] and in interneurons in the stratum radiatum of the hippocampus [31].

## 2. Some Neuroanatomical and Neurochemical Details of Peripheral Neurons and the Spinal Cord

### 2.1. Sensory and Autonomic Ganglia

Sensory impulses, including pain, originating in the surface of the body (e.g., the skin) or deeper structures (e.g., muscles, joints, and viscera) are transmitted to the spinal cord by way of peripheral nerves. These are contributed by thousands of axons produced by sensory neurons (also called primary afferent neurons) contained in the DRGs and cranial ganglia [3]. The great majority of studies analyzing the characteristics of primary afferent neurons focus on “nonvisceral” DRGs, more specifically the 4th and 5th lumbar (L4-5) DRGs, which typically innervate muscles and skin of the leg and foot by way of the sciatic nerve, both in rodents and humans [72].

In contrast, visceral organs are characterized for their innervation by two different “extrinsic” currents: (1) the spinal current, including the pelvic (PN) and the lumbar splanchnic (LSN) nerves [73, 74] and (2) the cranial current, contributed by the vagus nerve [75]. These two currents originate in “visceral” DRG and cranial ganglia neurons, identified by means of retrograde tracing from their peripheral nerve endings in thoracic, abdominal, and pelvic organs. In particular, the PN and the LSN innervating the colorectum or the urinary bladder in rat [73, 76] and mouse [73, 76, 77] carry axons derived from: (1) peripheral projections of thoracolumbar (TL; T8-L1) and lumbosacral (LS; L6-S1) DRG neurons [73, 78]; (2) postganglionic projections of sympathetic neurons contained in the lumbar sympathetic chain (LSC); and (3) sympathetic and parasympathetic neurons present in the “mixed” major pelvic ganglion (MPG) [79–81]. In addition, and unlike other tissues and organs in the body, the gut is also provided with its own “intrinsic” autonomic innervation. This includes sensory and motor autonomic neurons found in the myenteric and submucosal plexuses, from the esophagus to the anus, and collectively referred to as enteric neurons, creating an intrinsic neuronal network [81, 82]. 

In normal conditions, rodent nonvisceral DRG neurons express a multiplicity of neurotransmitters and receptors, often in different combinations, and are generally considered glutamatergic [83, 84]. Three main subpopulations of DRG neurons have been characterized, including: (1) large and medium-sized neurons expressing NF-200; (2) small and medium-sized neurons expressing the calcitonin gene-related peptide (CGRP) and termed “peptidergic”; and (3) small and medium-sized “nonpeptidergic” neurons expressing components of the receptor for the glial-derived neurotrophic factor and binding of isolectin B4 (IB4; from the plant *Griffonia simplicifolia* I) to neuronal glycoproteins and glycolipids [83, 84]. However, the “nonpeptidergic” term should only be applied to subpopulations lacking CGRP, since colocalization between IB4 and CGRP has been shown in rats [85] and mouse [86]. Interestingly, and highlighting the neurochemical complexity of DRG neurons, subpopulations of nonvisceral DRG neurons, lacking both neuropeptides and/or IB4 while expressing the noradrenergic marker tyrosine hydroxylase (TH) [87] or the neuropeptide tyrosine receptor type 2 (Y2R) [88], have also been described.

Several molecules involved in pain sensing (nociception) are expressed by nonvisceral DRG neurons, including the already mentioned CGRP, the transient receptor potential cation channel, subfamily V, member 1 (TRPV1) [89], the P2X purinoceptor 3 (P_2_X_3_) [90], or the sodium channel NaV1.8 [91]. Finally, other transmitter candidates in DRG neurons are the nucleotide adenosine-triphosphate (ATP) [92, 93], the “gaseous” transmitter nitric oxide [93, 94], and neurotrophic factors such as the glial- and the brain-derived neurotrophic factors [83].

Visceral DRG neurons, like nonvisceral ones, are classically described as being glutamatergic [14] and normally coexpress a variety of molecules, including neuropeptides [95]. Thus, CGRP is synthesized by rat [76, 96–98] and mouse [76, 77] visceral sensory neurons. TRPV_1_, P_2_X_3_, IB4 [73], and even TH [99] are also expressed by visceral sensory neurons. 

Autonomic neurons are morphologically characterized as sympathetic or parasympathetic, based on their expression of noradrenaline (using TH or the norepinephrine transporter type 1 for identification) or acetylcholine (using choline acetyltransferase or the vesicular acetylcholine transporter (VAChT) for identification), respectively. However, it should be noted that some sympathetic neurons have also been shown to coexpress acetylcholine and neuropeptides [80, 100–102]. Finally, different subpopulations of enteric neurons in the myenteric and submucosal plexuses typically express markers such as TH, VAChT, nitric oxide synthase, and several neuropeptides [103].

### 2.2. Spinal Cord

Peripheral stimuli processed by primary afferent neurons are transferred to the spinal cord by way of their central axons (dorsal roots). In this manner, they penetrate the gray matter, which is divided into 9 laminae, from dorsal to ventral, and an area around the central canal (area X), as originally characterized through analysis of the morphology and arrangement of Nissl-stained cell bodies in transverse sections of the cat spinal cord [104]. Neurons in each laminae are arranged in a complex but ordered manner, based on morphology, neurochemical composition, and specific sensory modalities [105–107]. Thus, lamina I, the most superficial of all, receives cutaneous A*δ*- and C-fibers [108–111] and muscle and articular afferents [112, 113], as well as visceral afferents [114–116]. Laminae II, subdivided into outer (IIo) with densely packed cells and inner (IIi) [117], receives predominantly unmyelinated C-fiber afferents [110, 118–120]. In addition, A*δ*-fibers terminate mostly in lamina IIo [121], and some cutaneous mechanoreceptive A*β*-fibers reach lamina IIi via lamina III [120, 122–125]. A*α*/*β*-fibers are the main afferent projections to laminae III–VI [110, 112, 123–129]. However, a subpopulation of fine C-fibers also penetrates deeply in the dorsal horn, into lamina III [98, 110, 112, 118, 130–132]. Finally, the area X receives a considerable input of visceral afferents [98, 115, 132–134], although terminals contributed by somatic afferents are also present [129, 135].

## 3. Expression of VGLUTs in Peripheral Visceral and Nonvisceral Glutamatergic DRG and Cranial Sensory Neurons

### 3.1. Somatic Expression of VGLUTs

**Figure 1:**
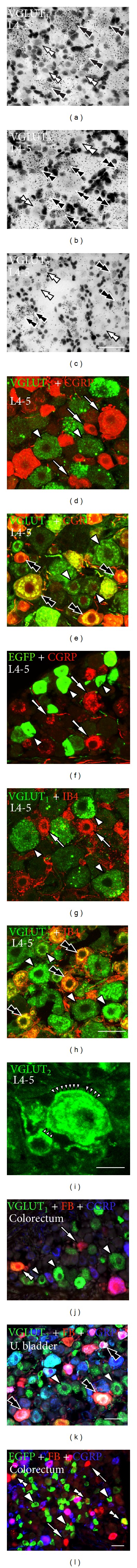
*Broad VGLUT expression in mouse visceral and nonvisceral DRG neurons*. Bright-field ((a)–(c)) and immunofluorescence ((d)–(l)) photomicrographs of DRG sections incubated with VGLUT_1_, VGLUT_2_, or VGLUT_3_ antisense riboprobes ((a), (b), (c), resp.) or VGLUT_1_ ((d), (g), (j)), VGLUT_2_ ((e), (h), (i), (k)), or EGFP ((f), (l)) antibodies (EGFP, used as a reporter of VGLUT_3_ expressing neurons). Colocalizations with CGRP ((d)–(f), (j)–(l)) or IB4 ((g), (h)) are also shown. Colorectum ((j), (l)) or urinary bladder (k) DRG neurons are exposed by presence of the retrograde tracer FB ((j), (k), (l)). ((a)–(c)) VGLUT transcripts are detected in nonvisceral DRGs, VGLUT_1_ mostly in large and medium-sized NPs (black double arrowheads in (a)), VGLUT_2_ in many NPs spanning different cell body sizes (black double arrowheads in (b)), and VGLUT_3_ in a discrete number of usually small and some medium-sized NPs (black double arrowheads in (c)). Many NPs lacking VGLUTs are also detected (white double arrowheads in (a)–(c)). ((d)–(f)) The distribution of VGLUT_1_- (arrowheads in (d)) VGLUT_2_- (arrowheads and black double arrowheads in (e)), and EGFP-IR (arrowheads in (f)) nonvisceral DRG NPs correlates with the transcript counterparts shown in (a)–(c). While most CGRP-expressing nonvisceral DRG NPs coexpress with VGLUT_2_ (black double arrowheads in (e)), VGLUT_1_-, VGLUT_3_- (arrowheads in (d), (f)), and CGRP-IR (arrows in (d), (f)) NPs virtually always appear as independent subpopulations. Many VGLUT_2_-IR DRG NPs lacking CGRP are also detected (arrowheads in (e)). ((g), (h)) IB4 is virtually always present in VGLUT_2_-IR nonvisceral DRG NPs (black double arrowheads in (h)), while many other VGLUT_2_-only NPs are also observed (arrowheads in (h)). In contrast, VGLUT_1_- (arrowheads in (g)) and IB4-expressing (arrows in (g)) DRG NPs always belong to different subpopulations. (i) VGLUT_2_-membrane staining is detected in a number of nonvisceral DRG NPs (small arrowheads). ((j)–(l)) VGLUT_1_- (arrowheads and with double arrowhead in (j)), VGLUT_2_- (arrowheads and black double arrowheads in (k)), and EGFP-IR (arrowheads and white double arrowheads in (l)) are detected in visceral DRG NPs, with a similar cell soma distribution as observed for nonvisceral DRGs. CGRP-IR visceral DRG NPs are virtually always VGLUT_2_-IR (black double arrowheads in (k)), while NPs expressing the peptide (arrows in (j), (l)) and VGLUT_1_ (arrowheads and double arrowheads in (j)) or EGFP-IR NPs (arrowheads and double arrowheads in (l)) belong to different subpopulations. Scale bars: 50 *μ*m ((c) = (a), (b); (h) = (d)–(g); (k) = (j); (l)); 20 *μ*m (i).* Data in figures (f) and (l) is previously unpublished, and the tissue has been processed according to the immunohistochemical procedures described in [54, 60–62].*

The expression of VGLUT_1_ mRNA in large and some medium-sized L4-5 DRG neurons was first shown (although not quantified) in the rat [48, 51]. Subsequent analysis in the mouse [53, 54] revealed presence of VGLUT_1_-IR neurons in 12% to 37% of nonvisceral DRG neurons (Figure 1, Table 1), while its transcript has been detected in ~45% of L4-5 DRG neurons of large and medium size [136]. Immunohistochemical signal for VGLUT_1_ is also observed in large and medium-sized visceral DRG neurons projecting to the mouse colorectum (12% [61]) and urinary bladder (32% [62]) (Figure 1, Table 1). Variations between the reported proportions of VGLUT_1_-expressing DRG neurons may depend on: (1) differences in protein and gene regulation between DRG levels (TL versus LS versus L4-5); (2) the use of different VGLUT_1_ antibodies/probes, immunohistochemical and *in situ* hybridization techniques or even mouse strains; and (3) especially when it comes to transcript versus protein mismatches, differential regulatory mechanisms.

In contrast to VGLUT_1_, VGLUT_2_ has been found in a large proportion of DRG neurons, spanning different cell soma sizes (Figure 1, Table 1), as initially shown in immunohistochemical studies in DRGs targeting the rat ileum [44] or *in situ* hybridization analysis of lumbar nonvisceral DRGs [51]. In the mouse, from ~65% [54] and up to 90% [137] VGLUT_2_-IR NPs are present in L4-5 DRGs. Even higher percentages of VGLUT_2_-IR NPs have been described in visceral DRG neurons innervating the colorectum [61] or the urinary bladder [62]. Confirming the abundance of VGLUT_2_ in DRG neurons, up to 70% [136] or 82% [137] of mouse L4-5 DRG neurons have been shown to express VGLUT_2_ mRNA.

Identifying VGLUT_3_ in peripheral sensory neurons has been more difficult than for the other VGLUTs, mainly due to the unavailability of antibodies that reliably labelled DRG neurons (in fact, this is still the case today). However, using transgenic mice expressing the enhanced green fluorescent protein (EGFP) under the control of VGLUT_3_ regulatory sequences, Seal et al. [66] showed that around 10-11% of nonvisceral lumbar DRG neurons express VGLUT_3_ [66]. This has been recently confirmed by *in situ* hybridization, with detection of VGLUT_3_ mRNA in ~17% of nonvisceral DRG NPs [65, 136], and through the identification of ~19% of transgenic adult mouse nonvisceral DRG NPs expressing the reporter gene Tomato, under the control of the VGLUT_3_ promoter [65] (Figure 1, Table 1). VGLUT_3_ is also present in subpopulations of visceral sensory neurons innervating both the colorectum (~10%, Figure 1, Table 1) or the urinary bladder (~18% [62]). VGLUT_3_ appears mostly expressed in small and medium-sized adult DRG NPs [62, 65, 66, 136]. However, a transient versus persistent expression of VGLUT_3_ has been proposed, where the transporter is found in large and medium-sized myelinated nonvisceral DRG neurons only during prenatal stages, with the small neuron population remaining VGLUT_3_-expressing during the adult life [65].

In DRGs projecting to visceral organs, an additional peculiarity is observed. Thus, TL and LS DRGs innervating the mouse colorectum [61] or the urinary bladder [62] contain different proportions of neurons expressing VGLUT_1_ or VGLUT_3_ (see Table 2), whilst VGLUT_2_ is equally abundant at both DRG levels [62]. Similar variations were also observed for other markers. For instance, the transient receptor potential cation channel, subfamily A, member 1 (TRPA_1_) mRNA is abundant in mouse TL bladder neurons but scarce in LS bladder neurons [138]. On the contrary, TH protein is expressed in threefold (colorectum) and fivefold (urinary bladder) greater proportions in mouse LS DRGs than in TL DRGs [62]. Interestingly, it has been postulated that differences in neurochemical expression between DRG levels could have an impact in the electrophysiological properties of TL and LS visceral afferent neurons [139, 140], as shown in the mouse colorectum, where a higher expression of TRPV_1_ in TL than in LS DRGs corresponds with a more robust response to applied capsaicin in colorectal TL nerve terminals [140].

The frequent association of VGLUT_2_-Li to the plasma membrane in nonvisceral [54] and visceral [61, 62] DRG neurons (Figure 1) has raised the question of if somatic glutamatergic release was possible in these neurons [54]. Accumulating evidence suggests that messenger molecules in general are released from the somatic compartment of the neuron [146], including DRG neurons [147–150]. Thus, neuropeptides [147, 148], ATP [149], and even genetically expressed macromolecular tracers in DRG neurons [150] have been shown to undergo somatic release. Interestingly, recent studies in *Xenopus* oocytes transfected with VGLUT_2_ suggest that this transporter can be found in two different states: (1) serving the traditional role of packaging glutamate into synaptic vesicles for Ca^2+^-dependent exocytosis, and (2) participating in Ca^2+^-independent leakage when present in the plasma membrane [151]. Moreover, this dual role appears to be also true for VGLUT_1_ and VGLUT_3_ [151]. Whether such states are also a feature in sensory neurons, and VGLUTs facilitated any form of somatic glutamatergic release remains to be established.

Finally, cranial sensory neurons are richly provided with VGLUTs, and their expression varies between target organs (Table 3).

### 3.2. Neurochemical Phenotype of VGLUT-Expressing DRG Neurons

**Figure 2:**
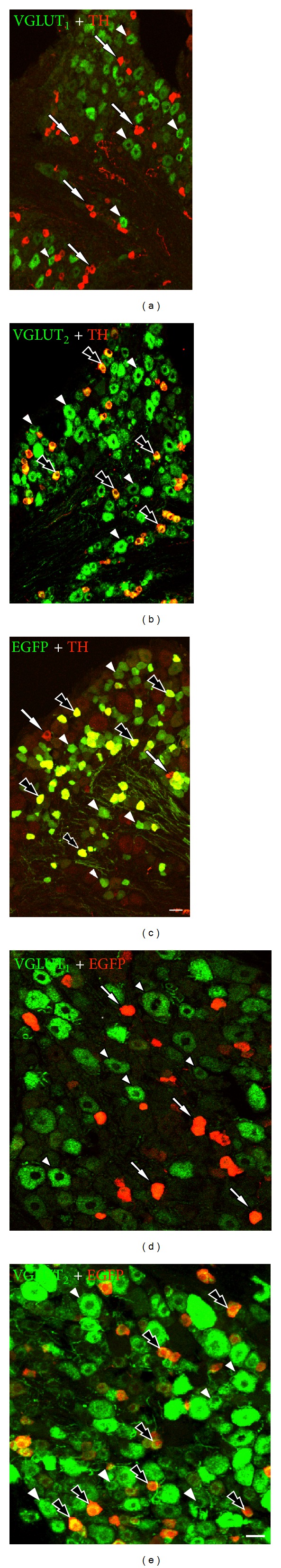
*Frequent coexpression of VGLUT*_*2*_ *or EGFP (VGLUT*_*3*_ *) with TH or VGLUT*_*2*_ *and EGFP in mouse nonvisceral DRG neurons*. Immunofluorescence photomicrographs of nonvisceral DRG sections incubated with VGLUT_1_ ((a), (d)), VGLUT_2_ ((b), (e)), or EGFP antibodies ((c), (d), (e)). ((a)–(c)) While virtually no VGLUT_1_-IR (arrowheads in (a)) DRG NPs coexpress with TH (arrows in (a)), most VGLUT_2_- or EGFP-IR NPs present with TH-Li (black double arrowheads in (b), (c)). Abundant VGLUT_2_- (arrowheads in (b)), EGFP- (arrowheads in (c)), and some TH-only (arrows in (c)) expressing NPs are also detected. ((d), (e)) Lack of coexpression is observed between VGLUT_1_ (arrowheads in (d)) and EGFP (arrows in (d)). In contrast, EGFP is virtually always coexpressed with VGLUT_2_ in DRG NPs (double black arrowheads in (e)). Many other VGLUT_2_-only expressing DRG NPs are also detected (arrowheads in (e)). Scale bars: 30 *μ*m ((c) = (a), (b); (e) = (d)). *Data in figures (a)–(e) is previously unpublished, and the tissue has been processed according to the immunohistochemical procedures described in [54, 60–62].*

As described in Section 2.1, DRG neurons can be divided into peptidergic and nonpeptidergic. VGLUT_1_-IR DRG neurons, either nonvisceral [48, 53, 54] or visceral [61, 62] appear to be nonpeptidergic and lacking IB4 (Figure 1, Table 4) or TH (Figure 2), as shown in rat [48] and mouse [53, 54, 61, 62]. Li et al. [46] and Alvarez et al. [50], however, have reported that VGLUT_1_ may be found in neuropeptide containing afferents terminating in the laminae I and II of the rat dorsal horn. This suggests that some peptidergic DRG neurons in the rat may synthesize low levels of VGLUT_1_, only detected in primary afferent terminals in the spinal cord but not in the larger cell bodies, where the immunohistochemical signal may be “diluted”.

 Sharing some similarities with VGLUT_1_, VGLUT_3_ is expressed in nonvisceral DRG neurons lacking CGRP, only in around 7% of those binding IB4 [66] (Figure 1, Table 4), but in most cases coexpressing with TH (Figure 2), the latter being typically detected in nonpeptidergic, nonvisceral DRG neurons [87]. Accordingly, in visceral DRG neurons targeting either the colorectum (Figure 1) or the urinary bladder [62], VGLUT_3_ has only been detected in the nonpeptidergic subpopulation (Table 4).

In contrast to VGLUT_1_ or VGLUT_3_, a considerable number of VGLUT_2_-IR DRG neurons coexpress CGRP or IB4 in nonvisceral [54] as well as visceral [61, 62] mouse DRGs (Figure 1, Table 4). Conversely, virtually all DRG neurons expressing CGRP or binding IB4 synthesize VGLUT_2_. This is in agreement with the considerable colocalization of VGLUT_2_ and IB4 previously described in primary afferents in the rat dorsal horn [45, 46, 49, 50] and mouse DRG neurons [53] and the previously shown colocalization of glutaminase with CGRP [152] and of glutamate-Li with CGRP or substance P in rat [11, 14, 153]. Accordingly, colorectal—[61] and urinary bladder-projecting [62] DRG neurons often coexpress VGLUT_2_ and CGRP (Figure 1, Table 4). Finally, virtually all VGLUT_2_-expressing nonvisceral DRG neurons in the mouse express TH (Figure 2), and in rat [154] and mouse [155], colocalization with TRPV1 has also been reported.

### 3.3. VGLUTs Colocalization in Cranial and DRG Neurons

It is now clear that at least some neurons in the nervous system express more than only one type of VGLUT. This was not the understanding when VGLUTs were first described. Thus, initial observations in the adult mammalian CNS showed a complementary distribution for VGLUT_1_ and VGLUT_2_ [26, 28, 30, 156, 156, 157]. Moreover, this complementarity seemed to extend to the synaptic level, where both VGLUTs appeared segregated in physiologically different synapses in the CNS [26, 30, 158, 159]. However, the finding of glutamatergic terminals in the rat hippocampus, containing both VGLUT_1_ and VGLUT_2_, suggested the possibility of a supplementary distribution [157].

Such a possibility is in fact also a feature in the periphery. Thus, in rat trigeminal ganglia [45] and nonvisceral DRGs [51], coexpression of VGLUT_1_ and VGLUT_2_ protein or mRNA has been reported in a subpopulation of neurons, respectively. This was confirmed in mouse (Table 4), were a moderate coexpression of VGLUT_1_ and VGLUT_2_ was shown in some medium-sized and large nonvisceral DRG neurons [54]. Coexpression of VGLUT_3_ and VGLUT_2_ (but not VGLUT_1_) is also detected in nonvisceral DRGs (Figure 2, Table 4). Finally, the overwhelming presence of VGLUT_2_ in virtually all colorectal [61] and urinary bladder [62] DRG neurons, indirectly implies a considerable degree of colocalization with VGLUT_1_ or VGLUT_3_.

How coexpression of two VGLUTs in the same neuron influences its role in neurotransmission is not yet known. It has, however, been suggested that VGLUT expression may be associated with different patterns of neurotransmitter release. Thus, VGLUT_1_ is normally expressed in CNS neurons with low probability release (climbing fibers in the cerebellum), whereas VGLUT_2_ would be associated to those with high probability (parallel fibers in the cerebellum) [160]. Whether the type of VGLUT expressed in a single DRG neuron or the coexpression of more than one VGLUT have an impact on release probability remains to be established.

### 3.4. VGLUTs in the Peripheral Projections of Cranial and DRG Neurons

VGLUTs in peripheral nerves were first identified in rat, mouse [161, 162], and guinea pig [163] esophageal intraganglionic laminar endings (IGLEs), dependent on vagal afferents, and shown to contain VGLUT_2_. VGLUT_1_ was also found in esophageal IGLEs in the guinea pig [163] and rat [164] but not the mouse [165]. Subsequent studies revealed presence of VGLUTs in peripheral nerves in many other locations in the upper body (Table 2).

**Figure 3:**
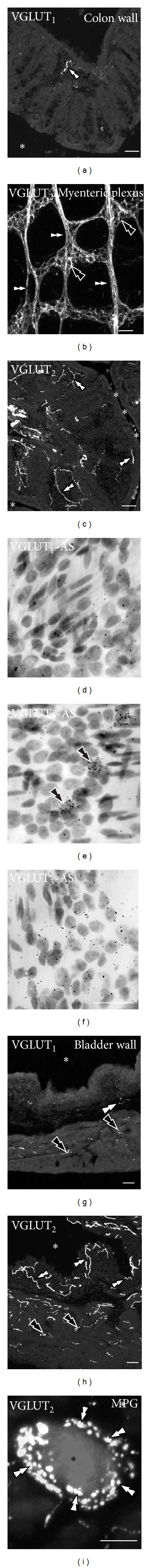
*Distribution of VGLUT-containing peripheral nerve fibers in mouse visceral organs and the MPG*. Immunofluorescence ((a)–(c), (g)–(i)) and bright-field ((d)–(f)) photomicrographs of sections of the colorectum ((a)–(c), (d)–(f)) the urinary bladder ((g), (h)) and the MPG (i), incubated with VGLUT_1_ ((a), (h)) or VGLUT_2_ ((b), (c), (h), (i)) antibodies or VGLUT_1_, VGLUT_2_ or VGLUT_3_ antisense riboprobes ((d), (e), (f), resp.). In (i), a colorectal MPG NP is exposed by its content of fast blue (asterisk). In ((a), (c), (g) and (h)), the position of the lumen is indicated by a white asterisk. ((a)–(c)) Isolated VGLUT_1_-IR nerve fibers are detected in the mucosal layer of the colorectum (double arrowhead in (a)), in contrast to the abundance of VGLUT_2_-IR nerve fibers in the myenteric plexus (black double arrowheads in (b)), the interconnecting nerve fibers (white double arrowheads in (b)), and the mucosal layer (white double arrowheads in (c)). ((d)–(f)) Only a number of VGLUT_2_ mRNA-expressing myenteric plexus NPs are detected in the mouse colorectum (black double arrowheads in (e)), in contrast to the absence of VGLUT_1_- or VGLUT_3_ mRNA-expressing NPs in such ganglia ((e), (f), resp.). ((g), (h)) While a small number of VGLUT_1_-IR nerve fibers are detected in the urinary bladder wall, especially in the muscular layer (black double arrowheads in (g)) and some in the submucosa (white double arrowhead in (g)), VGLUT_2_-IR nerve fibers are abundant and present throughout the whole thickness of the organ, including the muscular (black double arrowheads in (h)) and submucosal and mucosal layers (white double arrowheads in (h)). (i) A colorectal MPG NP is surrounded by a dense VGLUT_2_-IR basket (white double arrowheads). Scale bars: 50 *μ*m ((a)–(c), (g), (h)); 25 *μ*m ((d)–(f)); 10 *μ*m (i). *Figures (d) and (e) are reproduced in part, and with permission, from reference [61]. Data in figure (f) is previously unpublished, and the tissue has been processed according to the in situ hybridization procedures described in [60, 61].*

VGLUTs are also present in axonal terminations of abdominopelvic organs in the guinea pig [166] and mouse [61, 62, 167], where VGLUT_2_ appears to be the main player. Thus, VGLUT_2_-Li is found in abundant varicosities around VGLUT_2_-negative mouse colorectal myenteric plexus neurons (Figure 3) [61], in agreement with observations in the guinea pig [166] and mouse rectum [167], and supporting data on guinea pig small intestine showing glutamate-Li with a similar distribution [168]. VGLUT_2_ is also detected in an overwhelming number of nerve endings terminating in the urinary bladder (Figure 3) [62]. Unlike VGLUT_2_, VGLUT_1_ is only found in very few nerve fibers in the mouse colorectum [61], and in a small number of fibers in the urinary bladder, mostly within the muscular layers of this organ (Figure 3) [62]. Discrete VGLUT_1_ expression has also been reported in the Pacinian corpuscle and associated neurites in the cat mesentery [55]. The disparate representation of VGLUT_1_ and VGLUT_2_ in peripheral nerves terminating in visceral organs is supported by Olsson et al. [166], showing that ~3% of anterogradely traced guinea pig rectal nerve varicosities terminating in the myenteric plexus contain VGLUT_1_, whereas ~11% exhibit VGLUT_2_-Li.

**Figure 4:**
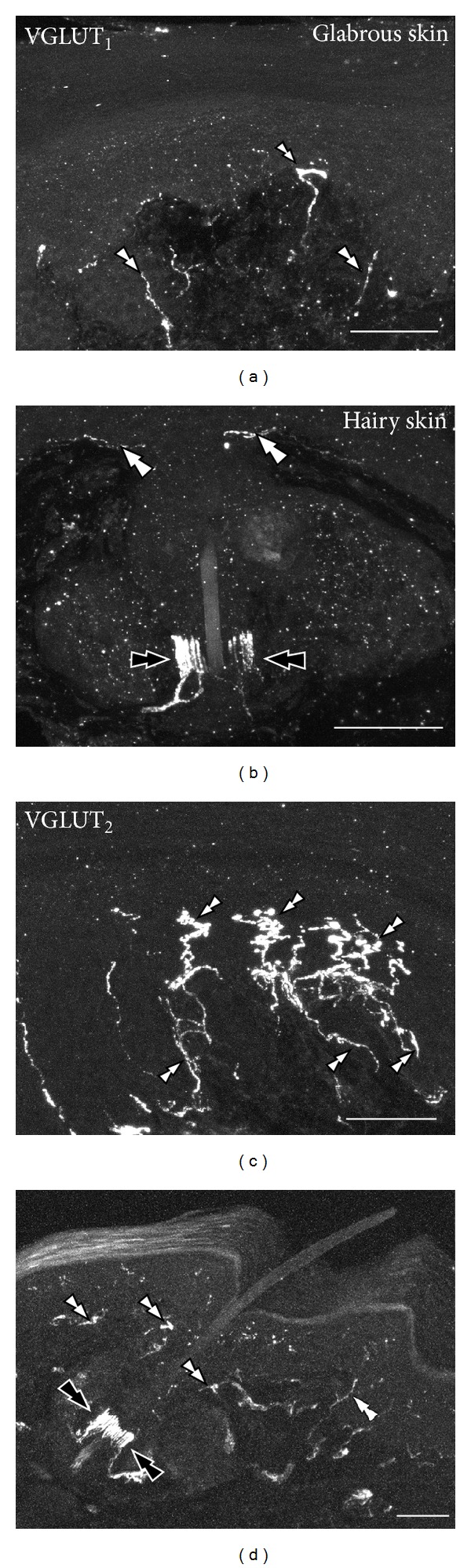
*Distribution of VGLUT-containing peripheral nerve fibers in the mouse skin*. Immunofluorescence photomicrographs of sections of the glabrous ((a), (c)) and hairy skin ((b), (d)) incubated with VGLUT_1_ ((a), (b)) or VGLUT_2_ ((c), (d)) antibodies. ((a), (b)) VGLUT_1_-IR nerve fibers are discretely observed in the glabrous (double arrowheads in (a), showing nerve fibers in close proximity to the epidermis) and the hairy skin (black double arrowheads in (b), showing the follicular neural network; white double arrowheads showing fibers lying in the basal membrane of the epidermis). ((c), (d)) Abundant VGLUT_2_-IR nerve fibers are detected in the glabrous (arrowheads in (c), showing nerve endings penetrating the epidermis) and the hairy skin (black double arrowheads in (d), showing the follicular neural network; white double arrowheads, showing nerve endings in the dermis and epidermis). Scale bars: 50 *μ*m ((c) = (a); (b); (d)). *Figures (b) and (d) are reproduced in part, and with permission, from [54].*

In the skin, the immunohistochemical presence of VGLUT_1_ and VGLUT_2_ has been studied in mouse [54] and rat [52, 169]. Thus, VGLUT_1_ is discretely expressed in dermal and epidermal nerves of the glabrous (hairless) skin, in the piloneural complex in hairy skin of mouse (Figure 4) [54], and in rat primary afferent endings in the muscle spindles in the triceps surae muscle [52]. Conversely, VGLUT_2_ is detected not only in piloneural complexes in hairy skin, but also in numerous nerve endings terminating in the glabrous hindpaw skin (Figure 4), both in deep dermal bundles as well as in close relation to the epidermis, often contacting VGLUT_2_-IR Merkel cells [54]. The presence of both VGLUT_1_- and/or VGLUT_2_-IR fibers in the piloneural complex suggests their origin in glutamatergic DRG neurons producing myelinated D-fibers [170].

Peripheral nerve endings containing VGLUT_3_ have been more difficult to analyze than those expressing VGLUT_1_ or VGLUT_2_, mainly due to lacking of reliable antibodies. However, free nerve endings in the mouse palatine mucosa expressing VGLUT_3_ (as well as VGLUT_1_ and VGLUT_2_) in addition to their presence in corpuscular nerve endings and Merkel cells, have been reported [171]. On the contrary, the limited number of VGLUT_3_-expressing colorectal [61] and urinary bladder [62] DRG neurons (identified in VGLUT_3_-EGFP mice [66]) suggests that only few if any nerve endings containing this transporter reach those organs. In skin, peripheral nerve endings produced by VGLUT_3_-expressing DRG neurons have been recently exposed by the use of transgenic mice where the reporter gene Tomato is expressed under the control of the VGLUT_3_ promoter [65]. In this study, VGLUT_3_-expressing DRG neurons were shown to terminate in the skin in two different fashions: (1) as C-low threshold mechanoreceptors forming longitudinal lanceolate endings around hairs, and (2) as epidermal free nerve endings [65]. The neuroanatomy of Tomato-positive fibers innervating visceral organs remains to be explored.

In accordance with the peptidergic nature of their parent DRG neurons, the great majority of nerve fibers innervating the colorectal mucosa in the mouse exhibit a high degree of coexpression of CGRP and VGLUT_2_ [61]. This is in contrast to nerve fibers located in the myenteric plexus, where most VGLUT_2_ and CGRP-IR fibers remained as different populations [61]. In support, nonpeptidergic VGLUT_2_-containing varicosities have also been reported in the esophageal myenteric plexus of rat [161]. Since a small subpopulation of VGLUT_2_-IR mouse colorectal DRG neurons is nonpeptidergic (~18%), it could be postulated that they selectively innervated the myenteric plexus. Alternatively, these nonpeptidergic VGLUT_2_-IR nerve fibers in the myenteric plexus could derive from neurons in the LSC or the MPG, two major contributors of nerve fibers in the colorectum [79] and the urinary bladder [172]. However, only rarely VGLUT_2_-IR neurons are observed in normal conditions in these ganglia [61, 62].

Finally, coexpression of VGLUTs in peripheral nerve endings has been shown for VGLUT_1_ and VGLUT_2_ in rat [164] and mouse [165] (but not in guinea pig [163]) IGLEs [173]. Also, Merkel cells in the rat sinus hair follicle express VGLUT_2_ and often show colocalization with VGLUT_1_ [169].

## 4. Expression of VGLUTs in the Spinal Cord

Thinly myelinated or unmyelinated low threshold A*δ*- and C-fibers transmitting nociceptive information and terminating predominantly in the superficial layers (laminae I and II) of the spinal dorsal horn release glutamate [174, 175]. Local spinal cord neurons are also capable of synthesizing and utilizing glutamate as their major excitatory neurotransmitter [3, 176, 177]. However, dissecting the patterns of expression and morphology of glutamatergic primary afferent terminals and spinal cord neurons from a “VGLUT perspective” has been challenging, mainly due to the failure of virtually all available VGLUT antibodies to produce immunohistochemical signal in the cell bodies of spinal cord neurons and the complex contribution of nerve terminals in the area (primary afferent versus local neurons, dendritic and axonal projections versus descending fibers). Nevertheless, the combined knowledge derived from immunohistochemical [30, 40, 46, 48–50, 54, 64, 66, 178–186] and *in situ* hybridization [43, 48, 51, 136, 187] studies allow today for a rather complete depiction of the VGLUT scenario in the spinal cord.

**Figure 5:**
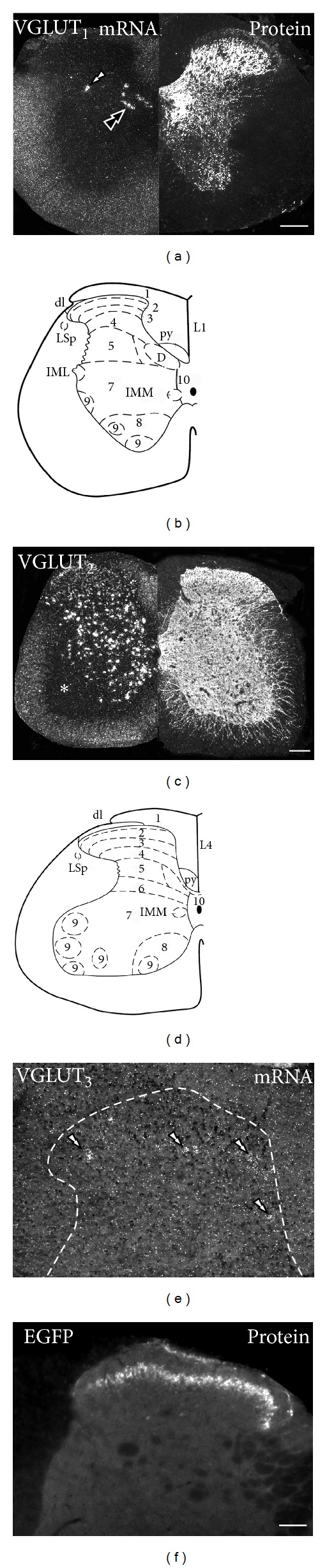
*VGLUTs protein and transcript expressions in the mouse spinal cord*. Dark-field ((a) and (c), left side; (e)) and immunofluorescence ((a) and (c), right side; (f)) photomicrographs of sections of the thoracolumbar and lumbar enlargement ((c), (e), (f)) of the spinal cord, incubated with VGLUT_1_, VGLUT_2_, or VGLUT_3_ antisense riboprobes ((a), (c), (e), resp.) or VGLUT_1_ (a), VGLUT_2_ (c), or EGFP (f) antibodies. Schematic drawings of the thoracolumbar (b) and lumbar enlargement (d) of the spinal cord are provided as references for the laminae in the gray matter (taken from The Rat Brain in Stereotaxic Coordinates, Fourth Edition, George Paxinos and Charles Watson, 1998). (a) A discrete number of VGLUT_1_ mRNA-positive NPs are detected in the dorsomedial aspect of the intermediate dorsal horn at thoracolumbar segments (black double arrowheads) and in more isolated fashion, in laminae IV-V of the dorsal horn (white double arrowhead). An abundant VGLUT_1_-IR neuropil is also detected in the dorsal and ventral horns, being more intense in the deep dorsal horn and in area X and only weak in laminae I-II. (c) Abundant VGLUT_2_ mRNA-positive NPs are detected in the lumbar enlargement of the spinal cord, encompassing both the dorsal and ventral horns. The VGLUT_2_ mRNA signal in NPs in laminae II-III appears somewhat more diffuse than in deeper laminae. The areas occupied by laminae IX are, however, devoid of VGLUT_2_-expressing NPs (asterisk). VGLUT_2_-Li is abundant in the neuropil in the whole gray matter. ((e), (f)) Only few VGLUT_3_ mRNA-positive NPs are detected in laminae III-IV of the dorsal horn (white double arrowheads in (e)). A modest EGFP-IR neuropil is also observed in laminae II (f). Scale bars: 200 *μ*m ((a), (c)) 50 *μ*m ((f) = (e)). *Figure (f) is reproduced in part, and with permission, from [66].*

Thus, VGLUT_1_- and VGLUT_2_-Lis are easily detected in the neuropil of the gray matter in the spinal cord, although both transporters appear distributed differently between laminae (Figure 5). In rat [30, 40, 46, 48, 50] and mouse [54], VGLUT_1_-Li is strong in laminae IIi–IV, medial laminae V/VI, dorsal lamina VII, and around lamina IX motoneurons. In contrast, VGLUT_1_-Li is weak in laminae I, IIo, the lateral part of lamina V, the lateral spinal nucleus, and lamina VIII. Regarding VGLUT_2_, studies in rat [30, 46, 48, 50] and mouse [54] exposed its abundant presence in laminae I and IIo, areas known to receive nociceptive fibers. Deeper in laminae IV-V, VGLUT_2_-Li appears moderate but progressively increases towards the ventral horns. As for VGLUT_3_, either through detection of weak VGLUT_3_ and stronger EGFP immunohistochemical signals in transgenic mice, presence of the transporter has been shown in the neuropil of laminae I–IIi neuropil (Figure 5) [66]. The question is, how are these different immunohistochemical patterns generated?

 DRG neurons are undoubted contributors of VGLUT_1_-containing nerve fibers in the spinal cord. Varoqui et al. [30] were the first in suggesting this and later confirmed by studies showing that transganglionically labelled primary afferent terminals in the dorsal horn of the spinal cord of rat expressed VGLUT_1_- or VGLUT_2_-Li [49]. Further support came from studies using dorsal rhizotomy, a procedure that completely blocks the central axonal transport of molecules produced by DRG neurons. Thus, dorsal rhizotomy dramatically, but not completely, reduces VGLUT_1_-LI in the ventral horn (and to a lesser extent also in the dorsal horn), both in rat [46, 48, 50] and mouse [54].

 However, the persistence of at least some VGLUT_1_-Li after dorsal rhizotomy suggests additional sources, including: (1) primary afferents expressing this transporter and entering the spinal cord at levels higher and lower to the lesion and travelling certain distances before contacting their second order neurons; (2) local dorsal horn neurons; (3) brainstem- [48, 178] and cortical-derived [26, 178, 179] nerve terminals. Supporting the local origin, oligo- and riboprobe, radioactive or nonradioactive *in situ* hybridization studies by Kullander et al. [187] in neonatal mice, and Oliveira et al. [48] and Llewellyn-Smith et al. [40] in adult rats, revealed a few large VGLUT_1_ mRNA-positive neurons in the dorsomedial part of the intermediate zone of the dorsal horn of the thoracic spinal cord, resembling dorsal nucleus of Clarke neurons. Studies in adult rats also suggested presence of VGLUT_1_ mRNA-positive neurons in the lamina I of the dorsal horn [51], as well as in motoneurons (Figure 5) [43, 51]. More recently, in a study in adult mouse, we confirmed the expression of VGLUT_1_ mRNA in a small group of neurons also resembling the dorsal nucleus of Clarke, and in occasional deep dorsal horn neurons at thoracolumbar levels [136]. Other spinal neurons, including motoneurons or superficial dorsal horn neurons, lacked VGLUT_1_ [136]. The presence of VGLUT_1_ in the dorsal nucleus of Clarke, known to be the origin of the spinocerebellar pathways [188], is supported by the detection of abundant VGLUT_1_-Li in nerve fibers terminating in the anterior and posterior zones of the cerebellum [180], normally receiving spinocerebellar mossy fibers [181].

With one exception in the rat showing an ipsilateral decrease [46], dorsal rhizotomy appears unable to alter the immunoreactivity of VGLUT_2_ in the dorsal horn of both rat [48, 50] and mouse [54]. This suggested that most if not all VGLUT_2_-Li was dependent on local or supraspinal neurons. In fact, supraspinal sources of VGLUT_2_ in the spinal cord have been recently demonstrated, as shown by their immunohistochemical presence in rubrospinal, vestibulospinal and reticulospinal tracts in rat [178]. However, a great proportion of VGLUT_2_-Li in the spinal cord is likely dependent on numerous spinal cord neurons, as shown by their expression of VGLUT_2_ transcript in the ventral and lateral aspects of the intermediate zone, in discrete parts of the ventral horns, and in the dorsal horn in rats [40, 48] and mice [136, 187] (Figure 5).

VGLUT_2_ mRNA-positive neurons in the spinal cord likely belong to both the interneuron [48, 49] and projection neuron subpopulations [40, 48, 51, 136]. On one hand, interneurons in the rat spinal cord of rat expressing somatostatin, neurotensin, substance P, and/or enkephalin coexpress VGLUT_2_ [49]. More recently, functionally identified excitatory interneurons in the rat have been shown to express VGLUT_2_-Li [177, 182, 183]. On the other hand, the presence of VGLUT_2_-Li in the large lemniscal and spinothalamic terminals to the ventral posterior thalamic nuclei in the rat [184] confirms that at least a number of neurons expressing VGLUT_2_ mRNA in the rat and mouse dorsal horn are projection neurons. Moreover, and as pointed out above, coexpression of both VGLUT_1_- and VGLUT_2_-Lis in mossy fibers in the cerebellum [180] indicates that Clarke's nucleus projection neurons also express VGLUT_2_. Finally, with the exception of one study in rat, suggesting that both VGLUT_1_ and VGLUT_2_ are expressed in motoneurons [43], other studies in rat [40, 48] and mouse [54, 136, 187] report that motoneurons, as well as neurons in area X, lack VGLUT_2_, at least at the lumbar enlargement. However, motoneurons express other glutamatergic markers such as glutamate itself [185, 189] and/or EAAT-3 [185] and thus may utilize a yet undescribed VGLUT.

Is then the contribution of primary afferents to the VGLUT_2_-Li in the dorsal horn of the spinal cord completely ruled out? The answer is no since VGLUT_2_ has indeed been identified in transganglionically labelled primary afferents in the dorsal horn [49], and it modestly accumulates after dorsal root ligation (DRG side of the ligation) [54]. It is thus possible that low quantities of VGLUT_2_ were transported by the central projections of DRG neurons and that the intense local- and supraspinal-dependent VGLUT_2_-Li in the spinal cord neuropil acted as a masking factor, potentially explaining lack of changes after dorsal rhizotomy [46]. Interestingly, the neuropeptide tyrosine receptor type 1 (Y_1_R), normally expressed by small primary afferent neurons, undergoes axonal transport and can be immunohistochemically detected in the dorsal horn, but its signal remains unaffected by dorsal rhizotomy [190]. In this case, and as discussed for VGLUT_2_, the abundant expression of the Y_1_R in local dorsal horn neurons appears to mask the expected decrease of the receptor after dorsal rhizotomy [190].

As for VGLUT_3_, its modest immunohistochemical detection in the superficial dorsal horn has been shown to depend on DRG and to a lesser extent also supraspinal and spinal cord neurons. Thus, (1) dorsal rhizotomy in transgenic mice results in an almost complete disappearance of VGLUT_3_-regulated EGFP-Li, normally observed in the superficial laminae of the dorsal horn, certifying its peripheral origin [66]; (2) Oliveira et al. [48], reported presence of a VGLUT_3_-IR neuropil in the sympathetic intermediolateral column (often coexpressing serotonin), supporting their origin in the dorsal and median raphe nucleus, where such localization has already been demonstrated [32, 33]; and (3) VGLUT_3_ protein [51, 136] and transcript [32, 51, 65, 136] have been demonstrated in the spinal cord of rat [32, 51] and mouse [65, 136], by means of RT-PCR [32] and western blot [51] in spinal cord extracts and by *in situ* hybridization in tissue sections [51, 65, 136]. More specifically, VGLUT_3_-expressing neurons have been detected in neurons in the deep dorsal horn and some in the ventral horn of adult rats [51], in the superficial and deep dorsal horn of neonatal mice [65], and in the deep dorsal horn of adult mice [136] (Figure 5). Interestingly, the VGLUT_3_-expressing subpopulation of neurons in the superficial layers of the dorsal horn described by Lou et al. in the neonatal mouse [65] is not detected in the adult mouse [136], suggesting developmental regulation of the transporter.

The complex peptidergic versus nonpeptidergic representation of VGLUTs in DRG neurons is also observed in their spinal axonal terminations. The general consensus is that VGLUT_2_ is often associated with peptidergic nerve terminals [46], whereas VGLUT_1_ is hardly so [48]. Thus, Li et al. [46] reported that SP-Li is present in ~50% of the VGLUT_2_-IR primary afferent boutons in laminae II [46]. In support, functional CGRP and AMPA receptors colocalize in single dorsal horn neurons, suggesting that these neurons may receive contacts from primary afferent terminals expressing peptides, glutamate, or both [191]. More importantly, AMPA receptor GluR_2_-IR puncta can be seen in contact with over 90% of CGRP-IR primary afferent synaptic boutons [192]. However, Todd et al. [49] reported that peptidergic primary afferents in the rat, as well as nonpeptidergic C-fibers, exhibit low levels of VGLUT_2_-Li or even lack either VGLUT_1_ or VGLUT_2_. 

Finally, and as observed in mouse DRG neuronal bodies [54], VGLUT_1_ and VGLUT_2_ colocalization is also detected in a proportion of primary afferent varicosities in laminae III-IV and IX in the rat spinal cord [49, 50, 179], as well as in the nucleus of the solitary tract [186]. The appearance of these varicosities is described as being “…relatively large… and contained immunoreactivity that was intense for VGLUT_1_ but weak for VGLUT_2_” [50].

## 5. Expression of VGLUTs in Autonomic Ganglia

### 5.1. Sympathetic and Parasympathetic Ganglia

In normal conditions, LSC neurons do not express VGLUTs [60–62], whereas only occasional VGLUT_2_-IR neurons are detected in naïve mouse MPG [61, 62]. However, VGLUT_2_-IR fibers are found in the mouse LSC and the MPG, in the latter forming perineuronal baskets (Figure 3) [60–62], often but not exclusively, around TH-IR MPG neurons [60].

 The VGLUT_2_-IR baskets observed in MPGs appear greatly dependent on extrinsic sources, as demonstrated by their dramatic immunohistochemical disappearance after axotomy of the pelvic nerve [60]. A sympathetic or parasympathetic preganglionic origin [80] for these VGLUT_2_-IR baskets has been ruled out due to their lack of coexpression with TH or VAChT, respectively. Alternatively, they could derive from primary afferent fibers in their way to pelvic organs and also running through the MPG [80, 193, 194]. In support, Aïoun and Rampin [195] have shown the ultrastructural coexistence of glutamate and large dense core vesicles; the latter typically loaded with peptides, in axons and terminals in the rat MPG. In the mouse, however, VGLUT_2_-IR MPG baskets lack CGRP [60]. Nevertheless, as described above, many nonpeptidergic mouse visceral DRG neurons express VGLUT_2_ [61, 62]. Whether VGLUT_2_-IR nonpeptidergic DRG neurons are both the origin of the MPG baskets, as well as of the neuropil surrounding myenteric plexus neurons in the mouse colorectum (see Section 3.4), and participate in sensory-motor coupling remains to be demonstrated. Finally, one additional source could be viscerofugal neurons projecting towards the MPG, found in rat [196], guinea pig [197], and mouse [198]. Interestingly, we recently showed a small subpopulation of myenteric neurons expressing VGLUT_2_ mRNA in the mouse colorectum [61] (see below).

### 5.2. Enteric Neurons

Most studies analyzing the expression of glutamate and glutamatergic markers in enteric neurons have focused on proximal rather than distal parts of the gut. Thus, enteric neurons containing immunohistochemically detectable glutamate have been described in the myenteric and submucosal plexuses of rat [168] and guinea pig ileum [168, 199], as well as in myenteric ganglia of the rat stomach [200]. More recently, a study in humans suggested that glutamate was present in large intestine submucosal and myenteric plexuses as well as in nerve fibers innervating the circular muscle layer [201], supporting earlier studies showing basal as well as stimulated (electrical and chemical) release of glutamate presumably from longitudinal muscle myenteric plexus neurons in the guinea pig ileum [202, 203].

As expected, VGLUTs are detected in enteric neurons in the gut. Thus, VGLUT_1_ was found in cholinergic and nitrergic neurons in rat [161, 164] and mouse [165] esophageal myenteric plexus. VGLUT_2_ was reported in neurons in the guinea pig, rat, mouse ileum [44, 204] and in rat [164] and mouse [162, 164] esophagus. Even in humans, all three VGLUTs appear to be present in the small intestine myenteric plexus neurons, interganglionic varicose fibers, and perisomatic puncta [205]. In the distal gut, lack of signal of VGLUT_1_ and VGLUT_2_ in enteric neurons of guinea pig rectum first suggested absence of glutamatergic enteric neurons, at least in this species [166]. In mouse, however, both protein and transcript of VGLUT_2_ are found in a small number of colorectal myenteric plexus neurons, scattered throughout the plexus in contrast to VGLUT_1_ [61] or VGLUT_3_ (Figure 3), which appear to be absent. What the phenotype of VGLUT_2_-expressing mouse myenteric plexus neurons is, remains to be established. However, glutamate and substance P or choline acetyltransferase colocalization was reported in small intestine enteric neurons of the guinea pig and rat [168].

## 6. Effects of Peripheral Nerve Injury or Tissue Inflammation on the Expression of VGLUTs

### 6.1. Sensory Ganglia and the Spinal Cord

Peripheral nerve injury [84, 207–209] as well as peripheral tissue inflammation [210–212] induces downregulation and upregulation of numerous molecules involved in a variety of functions that include survival, regeneration, and pain transmission in DRG and sympathetic ganglia neurons, as well as motoneurons in the spinal cord [84]. In line with such changes, Al-Ghoul et al. [213] reported an increase in the immunohistochemical levels of glutamate in the superficial layers of the dorsal horn after chronic loose ligation of the sciatic nerve, in parallel with the expected decrease of substance P and CGRP. Such increase could be related to alterations in VGLUTs synthesis and axonal transport.

In fact, peripheral nerve lesions alter the expression of VGLUTs in primary afferent neurons (Table 5). Thus, Hughes et al. [214] were the first in demonstrating that axotomy of the sciatic nerve in rats induces depletion of VGLUT_1_ protein in myelinated low threshold cutaneous and muscle mechanoreceptors terminating in the dorsal and ventral horns. It is now known that the depletion of VGLUT_1_ in the spinal cord is mainly due to its reduced expression in DRG neurons, as shown in mouse [54]. Axotomy of the sciatic nerve in the mouse also reduces the numbers of VGLUT_2_-IR DRG NPs, although a concomitant increase of VGLUT_2_-Li was detected in a subpopulation of small DRG NPs [54]. However, and in contrast to VGLUT_1_, changes in VGLUT_2_ expression in DRGs do not translate into expected decreases/increases in VGLUT_2_-Li at the lumbar levels of the spinal cord [54]. As explained for the lack of effect of dorsal rhizotomy on VGLUT_2_-Li in Section 4, such a “failure” could relate to what appears to be a modest transport of VGLUT_2_ from DRG neurons to the spinal cord [54], the abundant VGLUT_2_-expression by local dorsal horn neurons [48–50, 53, 54, 136] and the presence of the transporter in descending pathways [178]. A similar “failure” to detect changes after axotomy of the sciatic nerve was previously reported in rat for the Y_1_R in the superficial layers of the dorsal horn [215].

Somewhat surprisingly, the changes in immunohistochemical expression of VGLUT_1_ and VGLUT_2_ in DRG neurons appear to find no correlation in the expression of the corresponding transcripts. Thus, the number of VGLUT_1_- or VGLUT_2_ mRNA-positive DRG NPs remained unaltered in mice after a 7-day axotomy [136]. The only observed change was a modest downregulation in the number of VGLUT_3_ mRNA-positive DRG NPs [136], although it is not known if axotomy also alters its protein expression. It is possible that differences in the techniques (*in situ* hybridization versus immunohistochemistry) and mouse strains (BalbC versus NMRI mice) between studies explained the discrepancy. However, it is also possible that differences between transcript and protein regulations in DRG neurons after peripheral nerve injury had biological meaning (see Section 7).

Finally, in the only published account so far, inflammation of the hindpaw (using a unilateral intradermal injection of Complete Freund's Adjuvant) failed to induce changes in the expression of VGLUT_1_, VGLUT_2_, or VGLUT_3_ transcripts, both in DRG or spinal cord neurons [136]. Perhaps in these conditions, changes in expression and physiology of VGLUTs are more relevant in the axon and synaptic zones, where glutamate concentration and production is 2 to 3 times higher than in the cell body [19, 216, 217]. However, whether the inflammation of the hindpaw (or visceral organs) results in changes in VGLUT proteins in DRG neurons and their projections remains to be established.

### 6.2. Sympathetic Ganglia

Sympathetic neurons are profoundly affected by peripheral nerve injury [84, 101, 102, 209]. Thus, postganglionic axotomy of sympathetic nerves in cat LSC [218] or rodent superior cervical ganglion (SCG) [101, 102, 206] induces downregulation of neuropeptides such as CGRP [218] and NPY, as well as the noradrenergic marker TH [101, 102, 219, 220], and upregulation of galanin [101, 102, 218], VIP, SP [101, 102, 221], and the NPY Y_2_-receptor [101, 102, 206]. Also in pigs, the axotomy of colonic nerves containing sympathetic fibers projecting from the LSC, results in the upregulation of galanin and somatostatin, paralleled by the downregulation of TH [222].

**Figure 6:**
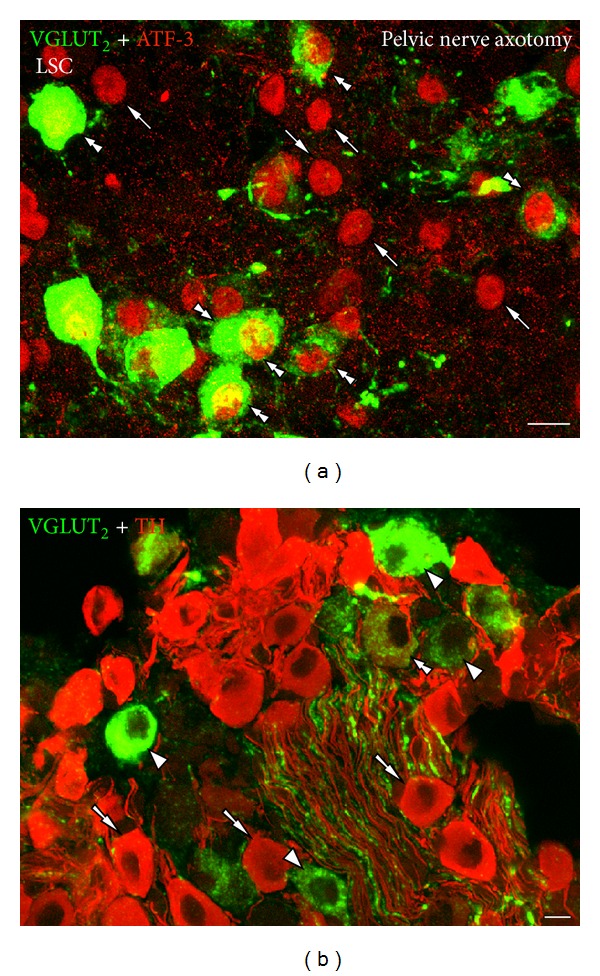
*VGLUT*_*2*_ *is upregulated in LSC neurons and occasionally coexpresses with TH*. Immunofluorescence photomicrographs of sections of the LSC of mouse after pelvic nerve axotomy, incubated with VGLUT_2_ ((a), (b)) and ATF-3 (a) or TH (b) antibodies. ATF-3 is used as a marker of injured neurons. (a) Pelvic nerve axotomy results in *de novo* expression of ATF-3 in an abundant number of LSC NPs (arrows). Pelvic nerve axotomy also results in *de novo* upregulation of VGLUT_2_, always coincidental with the upregulation of ATF-3 (double arrowheads). (b) Most VGLUT_2_-IR LSC NPs observed after lesion lack TH (arrowheads), although occasional VGLUT_2_/TH-IR NPs are detected (double arrowhead). Abundant TH-IR NPs are present in the LSC (arrows). Scale bars: 10 *μ*m.

Only until recently, glutamate was not thought to be present in autonomic neurons [81]. In fact, apart from a few VGLUT_2_-IR nerve fibers present in LSCs (more abundant in the stellate ganglion; unpublished results), VGLUTs are not normally expressed by neurons in these ganglia [60–62]. However, axotomy of either the pelvic (visceral) or the sciatic (nonvisceral) nerves results in the upregulation of VGLUT_2_ in a number of mouse LSC neurons (Table 5, Figure 6) [60]. The lesion-induced VGLUT_2_ plasticity appears to be selective in that parallel VGLUT_1_ protein and transcript or VGLUT_3_ mRNA upregulations are not observed [136]. The majority of VGLUT_2_-IR LSC neurons detected after injury lack TH, suggesting a parallel downregulation of the noradrenergic marker (see above). However, some LSC neurons upregulating VGLUT_2_ were shown to coexpress with TH, suggesting the possibility of glutamate and noradrenaline corelease [60] (Figure 6). Coexpression of VGLUT_2_ [223–228] or glutamate [223] with TH (used as a marker for dopaminergic neurons) has been previously shown in rat [223, 225–228] brainstem and hypothalamus [228]. Alternatively, VGLUT_2_ could be upregulated in LSC cholinergic (nonnoradrenergic) neurons [229], although this remains to be demonstrated.

The upregulation of VGLUT_2_ in LSC neurons could represent *de novo* synthesis of transporter protein and transcript, or an increase from low, undetectable levels. The absence of VGLUT_2_ (or other VGLUTs) transcript, using the sensitive riboprobe *in situ* hybridization techniques suggests the former hypothesis [60]. Considering the importance of VGLUTs for the uploading of glutamate into synaptic vesicles, it could be speculated that some LSC neurons, upon injury, acquire a glutamatergic phenotype, may release glutamate, and could potentially contribute to increased fast synaptic transmission and nociceptive mechanisms [60].

## 7. VGLUTs Modulation after Peripheral Nerve Injury—Implications to Glutamatergic Neurotransmission

While it is still not known exactly how many VGLUT copies are in each synaptic vesicle [35], it seems intuitive that changes in their expression after tissue insult should have profound consequences in glutamate loading. In fact, several studies where the expression of VGLUTs in synaptic vesicles is genetically manipulated suggest that the type and/or number of VGLUTs matter [19, 35]. For instance, the number of VGLUT copies in a given synaptic vesicle/neuron influences the amount and rate of vesicle loading, the size of glutamatergic quanta, and even the reserve pool of synaptic vesicles [158, 230–232].

In line with the above, the downregulation of VGLUT_1_ [214] and VGLUT_2_ proteins [54] observed in DRG neurons after sciatic nerve axotomy, while maintaining unaltered mRNA levels [136], may imply that these neurons sustain transcriptional levels of VGLUT_1_ and VGLUT_2_, in order to counteract the downregulation at the cell body level the latter resulting from increased axonal transport, protein use, and depletion at peripheral and central terminals [54]. Interestingly, axotomy of the sciatic nerve in rats results in a reduction in the number of synaptic vesicles in the central terminals of axotomized primary afferents [233], including peptidergic ones [234], suggesting increased fusion of clear synaptic vesicles (likely expressing VGLUTs) to the plasma membrane and glutamatergic release.

However, it should be noted that glutamatergic neurotransmission, from a synaptic vesicle point of view, is influenced by a variety of additional factors, comprising: (1) several steps to produce mature synaptic vesicles, mostly at the axons, although some may already happen in the soma [235]; (2) an active and tight regulation of presynaptic vesicle and transmitter recycling at the level of the synaptic cleft, to counteract depletion in situations of high activity [19, 216, 236]; (3) the extravesicular/cytoplasmic glutamate concentration (regulated by the enzyme glutaminase) 2 to 3 times higher in the terminals than in the cell body [217] and crucial in defining intravesicular glutamate content [19, 216]; (4) chloride conductance, along with the synaptic membrane potential, as also determining the glutamatergic content of synaptic vesicles and involving the participation of VGLUTs [237]; and (5) vesicular size, as it has been shown that there are different naturally occurring sizes that influence the quanta for different neurotransmitters, including glutamate [238]. How all these factors are challenged by peripheral nerve or tissue injury is not yet known. However, it has been shown that glutaminase is upregulated in rat DRG neurons during hindpaw inflammation and that its blockade results in reduced pain-like behaviour [239].

Altogether, nerve injury and/or inflammation and the accompanying pain, may result from changes in the expression of synaptic vesicles, associated proteins (including VGLUTs) and neuronal glutamatergic machinery in general, contributing to a fine “tuning” of pain mechanisms both at the synaptic as well as the cell body levels (see below).

## 8. VGLUTs, Proprioception, Pain, Survival, and Neuroprotection

### 8.1. Proprioception

Different studies in the peripheral and nervous systems suggest that proprioception is served by glutamatergic neurons expressing VGLUTs. This hypothesis is based on many of the observations in rodents presented above: (1) VGLUT_1_ is expressed by a number of large and medium-sized DRG neurons [48, 51, 54] likely producing myelinated peripheral projections terminating in muscle and joint proprioceptors; (2) VGLUT_1_-IR DRG neurons project fibers that have been morphologically characterized as proprioceptive [50, 214] towards the ventral horn and establish primary afferent-motoneuron contacts [40, 48–51, 54, 187, 214]; (3) many VGLUT_1_-IR (likely proprioceptive) fibers also terminate in the deep dorsal horn, in the area occupied by Clarke's nucleus [48, 50, 54, 136, 187]; (4) Clarke's nucleus, composed by the second-order neurons giving rise to the spinocerebellar proprioceptive pathway [188] expresses VGLUT_1_ [40, 48, 136, 187], and likely also VGLUT_2_ [136, 180]; (5) VGLUT_1_-Li (and also VGLUT_2_) is detected in mossy fibers terminating in the anterior and posterior zones of the cerebellum [180], known to receive input from the spinocerebellar pathway described above [181]. In addition, and suggesting a role in the cortical control of motoneurons, pyramidal cells in the neocortex express VGLUT_1_ mRNA [26], and numerous rat corticospinal tract nerve fibers terminating in the ventral horns exhibit VGLUT_1_-Li [178, 179].

### 8.2. Nonvisceral Pain

The use of transgenic mice has exposed a central role for VGLUT_2_ in the glutamatergic mechanisms associated to nonvisceral pain. Thus, heterozygote VGLUT_2_ knock-out (KO) mice (homozygote mice experience perinatal death) exhibit impaired mechanical and cold allodynia after spared sciatic nerve injury, despite maintaining normal acute pain responses and increases in pain-like behaviour after inflammation of the hindpaw [240, 241]. Such behavioural patterns after partial VGLUT_2_-KO originally suggested the involvement of the thalamus, whose neurons are richly provided with VGLUT_2_ and presented with altered electrophysiological function in the transgenic mice [241]. However, mechanisms involving VGLUT_2_ and pain appear to be relevant also at the DRG level. Thus, the selective deletion of VGLUT_2_ in a subpopulation of TH- and TRPV_1_-expressing neurons in mouse DRGs resulted in increased itch and decreased thermal pain sensitivity [155]. Interestingly, peripheral nerve injury results in VGLUT_2_-Li increases in small mouse DRG neurons [54] (see Section 6.1), and it is possible that these were TRPV1-expressing (see Section 3.2). Thus, a hypothesis could be that activation of TRPV_1_ in neurons upregulating VGLUT_2_ during peripheral neuropathy may contribute to heightened peripheral and/or central release of glutamate, in the latter, resulting in the activation of nociceptive second-order projection neurons present in laminae I-II [242].

 Selective deletion of VGLUT_2_ in another subpopulation of DRG neurons expressing the sodium channel subtype Nav1.8 resulted in altered pain responses, including attenuated responses to intense mechanical stimuli [243]. Interestingly, most of these effects seem to be associated with reductions in glutamate release in the superficial layers of the dorsal horn, as shown by the reduced c-fos activation of local dorsal horn neurons upon noxious heat stimulation [155] or intraplantar injection of capsaicin [244]. Moreover, a reduction in spontaneous excitatory postsynaptic currents was observed in lamina II dorsal horn neurons in the VGLUT_2_-KO-Nav1.8 mice [244].

Of the other known VGLUTs, VGLUT_1_ appears mostly associated with proprioception (see above) and the transmission of tactile stimuli, as it has been proposed that many VGLUT_1_-expressing primary afferent terminals in the superficial and deep dorsal horn correspond to low-threshold cutaneous mechanoreceptors, including those associated with the piloneural complex, in particular for nerve fibers terminating in lamina IIi of the superficial dorsal horn [49, 50, 214]. Deletion of VGLUT_1_ in mice has no effect on nonvisceral pain behaviour [240]. 

On the contrary, VGLUT_3_ is currently the center of debate. On one hand, deletion of VGLUT_3_ in mice results in an increased threshold to intense noxious mechanical stimuli and reduced mechanical hypersensitivity to normally innocuous stimuli after tissue inflammation or nerve injury [66]. On the other hand, mice with deleted VGLUT_3_ through knockout of the *runt* domain transcription factor Runx1, essential for the developmental control of unmyelinated sensory neurons (nociceptors, pruriceptors, and thermoceptors) [245] and also VGLUT_3_ in sensory neurons [65], did not show major changes in acute and chronic mechanical pain, with the exception of a modest increase in mechanical threshold after hindpaw carrageenan injection [65]. However, Seal et al. [66] utilized mice with global VGLUT_3_ knockout (these mice also having deafness and rare nonconvulsive seizures), and thus, the behavioural effects observed in their study could also be influenced by deletion of the transporter in the spinal cord or other parts of the brain. With some limitations as well, the study by Lou et al. [65] analyzed neurons lacking VGLUT_3_ but also Runx1, the latter influencing the expression of additional molecules in sensory neurons. Therefore, more research is needed to better understand the role of VGLUT_3_ in acute and chronic nonvisceral pain.

Finally, the role of VGLUTs in nonvisceral nociception may also extend into the peripheral projections of DRG neurons. This is suggested by the increased glutamate (but not aspartate) levels in the hindpaw extracellular space, upon A- and/or C-fiber stimulation of the sciatic nerve, as well as the local injection of kainate or capsaicin [246]. Such a release of glutamate results in depolarizing effects on primary afferent C-fibers and the induction of pain-related behaviour of exogenously applied glutamate [246–249], likely acting on presynaptic glutamate (auto) receptors of various types [250–254]. The type of VGLUT involved in these mechanisms has not been defined. However, it is possible that VGLUT_2_ was a main player, as suggested by its abundance in peripheral nerve endings in the skin [54].

### 8.3. Visceral Pain

The involvement of VGLUTs in visceral pain remains to be elucidated. The only study on the role of VGLUT_2_ in visceral pain published so far found no differences between VGLUT_2_-KO and littermate mice [240]. This is curious, especially when considering that the abundant numbers of VGLUT_2_-expressing colorectal [61] and urinary bladder [62] neurons imply the likely colocalization of the transporter with several molecules associated with nociception, such as TRPV_1_ [89], P_2_X_3_ [90], or the sodium channel NaV1.8 [91], only to cite a few. Thus, TRPV_1_ is abundantly expressed in rat and mouse colorectal DRG neurons [76, 255] and has been associated to mechanisms of chronic visceral pain [256], and coexpression of VGLUT_2_ with TRPV_1_ has been previously reported in nerve fibers terminating in the mouse rectum [167]. In addition, P_2_X_3_, implicated in nociception [257], particularly in visceral organs [258, 259], is also expressed by a proportion of colorectal DRG neurons [77], and a role for Na(v)1.8 in visceral pain and hyperalgesia has also been reported [260]. Therefore, it would be expected that deletion of VGLUT_2_ in so many neurons clearly prepared for nociception resulted in altered pain mechanisms. More research will be necessary to establish the extent to which VGLUT_2_ participates in the physiopathology of visceral disorders associated with discomfort and pain.

Finally, preliminary experiments suggest that VGLUT_3_ may not be involved in visceral hypersensitivity since its deletion does not alter the response to noxious mechanical distension of the colorectum, as compared to control mice (unpublished results). Accordingly, only a small percentage of VGLUT_3_-expressing DRG neurons innervating the urinary bladder [62] or the colorectum (Figure 1) have been identified. However, an association between changes in the expression of VGLUT_3_ in DRG neurons and visceral hyperalgesia in rats with *Trichinella Spiralis* infection has been proposed [64].

### 8.4. VGLUT-Expressing Sympathetic Neurons: Implications to Pain

The upregulation of VGLUT_2_ in sympathetic neurons in the LSC (see Section 6.2) positions them as the new “kid on the block”, acting as one additional contributor to peripherally released glutamate [60], along with primary afferents [48, 51, 53, 54, 54, 61, 62, 66, 154], and participating in processes of sympathetic-sensory neuron coupling [261, 262], also through glutamatergic neurotransmission.

In such scenario, glutamate released from primary afferent nerve terminals could act onto various types of glutamatergic receptors present in sympathetic postganglionic nerves [263–266], promoting an augmented release of noradrenaline and perhaps also glutamate. In turn, such “sympathetically” released glutamate could act on existing glutamatergic receptors in both visceral [267–270] and nonvisceral [175, 253, 271–275] primary afferent neurons, perpetuating a state of excitation in conditions such as inflammation [266] or nerve injury. More research will be necessary to: (1) assess the role of nerve injury-induced upregulation of VGLUT_2_ in LSC neurons; (2) explore if its expression is also affected by other types of visceral organs pathological conditions (e.g., ulcerative colitis or interstitial cystitis); and (3) what is the consequence of deleting VGLUT_2_ on autonomically driven nociceptive mechanisms.

### 8.5. VGLUTs, Survival, and Neuroprotection

The increased expression of VGLUT_2_ in LSC neurons appears tightly related to the occurrence of axonal damage, as shown by the concomitant upregulation of the activating transcription factor 3 (ATF-3) [60] (Figure 6), a classical marker of damaged peripheral axons [276]. Axotomy of the rat or mouse superior cervical ganglion postganglionic axons also results in *de novo* ATF-3 expression [277], although it is not known if VGLUT_2_ is upregulated in these neurons. Interestingly, ATF-3 [278], as well as the classical nerve growth factor (NGF), are central to mechanisms of nerve regeneration, and neuronal survival [80, 209]. Whether the upregulated VGLUT_2_ (and potentially also glutamate) had a role in the survival and regeneration of LSC neurons remains to be established. However, several studies support such concept. Thus, VGLUT_2_-Li is present in neurons migrating from the olfactory placode towards the forebrain in the developing rat brain, gradually decreasing toward adulthood [279]. Moreover, an association between the expression of VGLUT_2_ protein in mesencephalic dopaminergic (DA) neurons and their formation of synaptic junctions in the nucleus accumbens was demonstrated in rat [280]. Furthermore, conditional knockout of VGLUT_2_ results in reduced growth and survival of mesencephalic DA neurons, decrease in the density of DA innervation in the nucleus accumbens, reduced activity-dependent DA release, and impaired motor behaviour [281]. Thus, despite the established concept that excessive activation of glutamatergic receptors results in neurotoxicity [282], in certain cases glutamate could have the opposite effect and contribute to survival and neuroprotection [283].

 Interestingly, VGLUTs may also serve a role in development and neuroprotection in DRG neurons, as suggested in a recent study showing that glutamate release is essential to the development, maintenance, and sensory function of the piloneural mechanoreceptor, with VGLUT_2_ being a key player [284].

## 9. Could VGLUTs Become Pharmacological Targets for the Control of Pain?

Throughout this review, we have highlighted the current knowledge on VGLUTs in peripheral neurons and the spinal cord, their regulation by tissue injury, and their involvement in sensation and pain. The abundance of VGLUT_2_ in the periphery implies a fundamental role in glutamatergic physiology, even though the more discrete expression of VGLUT_1_ and VGLUT_3_ also suggests specific roles in select groups of DRG neurons. Data in transgenic mice, where a 50% reduction in VGLUT_2_ [155, 240, 241, 243] or total ablation of VGLUT_3_ [66] protein results in reduced/attenuated mechanical and cold hyperalgesia/allodynia after peripheral nerve injury or nonvisceral inflammation, while leaving unaffected other types of sensory processing, including acute nociception and inflammatory hyperalgesia, are compelling. Based on this knowledge, pharmacological blockade (total or partial) of VGLUT activity could efficiently reduce the amount of glutamate per vesicle, affect the size of glutamatergic quanta [241, 285, 286], and thus, attenuate glutamatergic neurotransmission, both at central and/or peripheral sites, resulting in the reduction of pain.

Exogenous VGLUT inhibitors, such as Chicago sky blue 6B (CSB6B), have been shown to inhibit the loading of glutamate into synaptic vesicles upon intracerebroventricular application [287], resulting in the inhibition of the methamphetamine induced hyperlocomotion and behavioural sensitization [288]. Interestingly, in an older study, Beirith et al. [289] evaluated the role of a systemically delivered CSB6B in animals receiving an intraplantar injection of glutamate and found that the use of the VGLUT inhibitor results in a considerable reduction of glutamate-induced nociception. However, the site of action of systemically applied CSB6B was not evaluated in that study, and thus, whether the inhibition of vesicular glutamate uptake occurs at peripheral nerve endings, spinal cord, supraspinal levels, or all of them remains to be established. Other exogenous compounds, including the dye Evans Blue or the Bengal Rose extract, have been described as VGLUT inhibitors and await further characterization [290, 291].

Endogenous VGLUT regulators also exist. Thus, fasting or diets with high lipidic and low glucose content (ketogenic diet), originally used to successfully reduce epileptic seizures [292], result in reduced pain and inflammation in juvenile and adult rats [292, 293]. Interestingly, the mechanisms of action proposed for ketogenic diets include decreases in the intracellular glutamatergic pool, as shown in cultured cerebellar granule neurons [294] and of glutamate uptake in synaptic vesicles by interference with the VGLUT chloride binding sites described in Section 7 [295]. However, the possible association of ketogenic diets, VGLUTs modulation, glutamate vesicular loading and pain mechanisms remain to be further established. Finally, different products of the kynurenine pathway in the metabolism of the amino acid tryptophan have been shown to exert antinociceptive roles after intraperitoneal administration in rats [296, 297], possibly through inhibition of VGLUTs activity [298].

Synthetic VGLUT inhibitors have been recently developed and their blocking action demonstrated *in vitro* [299, 300]. Unfortunately, these inhibitors do not discriminate between VGLUT types, and their role *in vivo* has yet to be determined. The development of selective VGLUT antagonists not only could help in dissecting the role of each VGLUT in *in vitro* and *in vivo* studies, but could also be therapeutically interesting, since regulation of the quantal size before fusion to the plasma membrane is emerging as an attractive approach to regulate the function of several neurotransmitters and as a tool to generate new pharmacological compounds [19, 301]. It is noteworthy that commercially available anticonvulsant agents such as gabapentin, lamotrigine, and riluzole limit glutamate release as part of their mechanisms of action and have been shown to be effective in reducing hyperalgesia in rats with neuropathy [302]. However, whether these agents act on VGLUTs to affect glutamate release is currently unknown.

 The reduction of pain behaviour by topical targeting of peripheral glutamatergic mechanisms should also be considered. Thus, blockade of peripheral glutamatergic receptors emerge as an interesting therapeutic option [253, 270, 274, 303, 304], especially in view of the complex and serious CNS-driven side effects of systemically delivered glutamatergic receptor antagonists [303, 305, 306]. Likewise, inhibiting the peripheral synthesis of glutamate by targeting glutaminase bears promise [16]. In fact, glutaminase is upregulated in rat DRG neurons during inflammatory processes of the hindpaw [239], and its peripheral inhibition results in a reduction of the inflammation-induced hindpaw edema and of c-fos expression in laminae I-II of the dorsal horn of rats, as well as long-lasting analgesia [307]. Finally, and based on their abundant peripheral representation (especially for VGLUT_2_), it is likely that challenging the activity of peripheral VGLUTs should also result in efficient modulation of glutamatergic neurotransmission.

## 10. Summary

In conclusion, this review has addressed various aspects relating to VGLUTs in visceral and nonvisceral DRGs, sympathetic neurons, and the spinal cord. When focusing on some of the functions of VGLUTs, the expression of VGLUT_1_ in primary afferent nerves terminating in spinal areas such as those occupied by the dorsal nucleus of Clarke (also VGLUT_1_-expressing) and motoneurons suggests a role in proprioception, whereas VGLUT_2_, and to some extent VGLUT_3_, exhibits a robust association to nociception and pain. Moreover, the frequent coexpression of VGLUT_2_ and CGRP supports the idea of corelease, and this could be relevant in processes of neurogenic inflammation. The *de novo* expression of VGLUT_2_ in the LSC supports previously unexpected roles, such as sympathetic glutamatergic neurotransmission and/or survival and neuroprotection. Finally, the efficacy of the genetic deletion of VGLUT_2_ (even if only half of what a neuron normally produces) and VGLUT_3_ for the control of pain in rodents highlights the potential of these VGLUTs as potentially interesting targets for the development of new analgesic compounds. In such line of thought, it would be important to analyze the presence and distribution of VGLUTs in human peripheral nervous tissue and how do they react to tissue or nerve insult.

## Figures and Tables

**Table 1:** Percentage of DRG neurons (visceral or nonvisceral) expressing VGLUTs (protein and mRNA).

VGLUT	Species	Percent nonvisceral afferents	Percent visceral afferents (target organs)
VGLUT_1_ protein	*Mouse *	~37% [53]; ~12% [54]	~12% (Colorectum [61]) ~32% (U. Bladder [62])
	*Rat *	Detected, not quantified [48]	NE (Colorectum, U. Bladder)

VGLUT_1_ mRNA	*Mouse *	~40% [136]	NE (Colorectum, U. Bladder)
	*Rat *	Detected, not quantified [48]	NE (Colorectum, U. Bladder)

VGLUT_2_ protein	*Mouse *	~65% [54]	~97% (Colorectum [61]) ~94% (U. Bladder [62])
	*Rat *	NE	Detected, not quantified (Stomach and Ileum [44]) NE (Colorectum, U. Bladder)

VGLUT_2_ mRNA	*Mouse *	~70% [136]	NE (Colorectum, U. Bladder)
	*Rat *	NE	NE (Colorectum, U. bladder)

VGLUT_3_ Protein	*Mouse *	~10% [66]; ~19% [65]	~10% (Colorectum*) ~18% (U. Bladder [62])
	*Rat *	NE	NE (Colorectum, U. bladder)

VGLUT_3_ mRNA	*Mouse *	~17% [136]; ~16% [65]	NE (Colorectum, U. Bladder)
	*Rat *	NE	NE (Colorectum, U. Bladder)

NE: not evaluated; *unpublished data; tissue has been processed and NPs quantified according to references [54, 60–62].

**Table 2:** Percentage of colorectal or urinary bladder DRG neurons expressing VGLUTs protein.

Target organ	DRG level	VGLUT_1_	VGLUT_2_	VGLUT_3_
Colorectum	Thoracolumbar	~15% [61]	~98% [61]	~17%*
	Lumbosacral	~8% [61]	~97% [61]	~4%*
Urinary bladder	Thoracolumbar	~39% [62]	~94% [62]	~28% [62]
	Lumbosacral	~26% [62]	~94% [62]	~8% [62]

*Unpublished data; tissue has been processed and NPs quantified according to references [54, 60–62].

**Table 3:** Presence of VGLUTs protein in cranial sensory neurons and their projections (upper part, VGLUTs presence in retrogradely traced cranial sensory neurons; lower part, immunohistochemical detection of VGLUTs in nerve terminals of cranial sensory neurons).

Organ/species	Cranial ganglia	VGLUT_1_	VGLUT_2_	VGLUT_3_
Rat stomach [44, 141]	Nodose	+	+	NE
Rat aortic depressor nerve [141, 142]	Nodose	Not detectable	+	+
Guinea pig trachea [58]	Nodose	+	+	NE
Mouse tongue [143]	Geniculate	+	+	NE
Rat cornea [45]	Trigeminal	+	+	NE

Organ/species	Nerve/terminals	VGLUT_1_	VGLUT_2_	VGLUT_3_

Rat pleura [56]	Laminar endings	+	+	NE
Rat heart [141]	Cardiac vagal afferents	+	Not detectable	Not detectable
Guinea pig trachea [58]	Cough mechanoreceptors	+	+	NE
Mouse tongue [143]	Taste Buds	+	+	NE
Rat masseter muscle [144]	Mesencephalic projections	+	NE	NE
Rat cornea [59]	Trigeminal	+	+	NE
Human teeth [63]	Trigeminal	+	+	NE
Rat lung [145]	Pulmonary neuroepithelial bodies	NE	+	NE

+: present; NE: not evaluated.

**Table 4:** Percentage of DRG neurons (visceral or nonvisceral) expressing VGLUTs and CGRP, IB4, or other VGLUTs.

Colocalization	Species	Percent nonvisceral afferents	Percent visceral afferents (target organ)
VGLUT_1_ also CGRP	*Mouse *	Not detectable [53, 54]	Not detectable (Colorectum [61], U. Bladder [62])
	*Rat *	Not detectable [48]	NE (Colorectum, U. bladder)

VGLUT_1_ also IB4	*Mouse *	Not detectable [53, 54]	NE (Colorectum, U. Bladder)
	*Rat *	NE	NE (Colorectum, U. Bladder)

VGLUT_2_ also CGRP	*Mouse *	~31% [54]	~81% (Colorectum [61])
	*Rat *	NE	~53% (U. Bladder [62])

VGLUT_2_ also IB4	*Mouse *	~42% [54]	NE (Colorectum, U. Bladder) NE (Colorectum, U. Bladder)
	*Rat *	NE	NE (Colorectum, U. Bladder)

VGLUT_3_ also CGRP	*Mouse *	Not detectable [66]	Not detectable (Colorectum*, U. Bladder [62])
	*Rat *	NE	NE (Colorectum, U. Bladder)

VGLUT_3_ also IB4	*Mouse *	~7% [66]	NE (Colorectum, U. Bladder)
	*Rat *	NE	NE (Colorectum, U. Bladder)

VGLUT_2_ also VGLUT_1_	*Mouse *	~14% [54]	Highly likely (Colorectum [61], U. Bladder [62])
VGLUT_2_ also VGLUT_3_	*Mouse *	~100%*	Highly likely (Colorectum [61], U. Bladder [62])
VGLUT_1_ also VGLUT_3_	*Mouse *	Not detectable*	NE (Colorectum, U. Bladder)

VGLUT_2_ also VGLUT_1_ VGLUT_2_ also VGLUT_3_ VGLUT_1_ also VGLUT_3_	*Rat *	NE	NE (Colorectum, U. Bladder)

NE: not evaluated; *unpublished data; tissue has been processed and NPs quantified according to references [54, 60–62].

**Table 5:** Changes in the expression of VGLUTs in DRGs, spinal cord, and/or LSCs, upon peripheral nerve injury (axotomy of the sciatic nerve) or hindpaw inflammation.

Tissue	Species	Lesion type	Protein			mRNA		
			VGLUT_1_	VGLUT_2_	VGLUT_3_	VGLUT_1_	VGLUT_2_	VGLUT_3_
DRG	*Mouse *	Axotomy	*▼▼* [54]	*▼* [54]^*α*^	NE	No change [136]	No change [136]	*▼* [136]
		Hind. inflam.	NE	NE	NE	No change [136]	No change [136]	No change [136]
	*Rat *	Axotomy	NE	NE	NE	NE	NE	NE
		Hind. inflam.	NE	NE	NE	NE	NE	NE

Spinal cord	*Mouse *	Axotomy	*▼▼* *▼* (LII-VIII, IX) [54]	No change [54]	NE	No change [136]	No change [136]	No change [136]
		Hind. inflam.	NE	NE	NE	No change [136]	No change [136]	No change [136]
	*Rat *	Axotomy	*▼▼* *▼* (LII-VIII-IX) [214]	NE	NE	NE	NE	NE
		Hind. inflam.	NE	NE	NE	NE	NE	NE

LSC	*Mouse *	Axotomy	No change [60]	▲ [60]	NE	No change [60]	▲ [60]	No change*
		Hind. inflam.	NE	NE	NE	NE	NE	NE
	*Rat *	Axotomy	NE	NE	NE	NE	NE	NE
		Hind. inflam.	NE	NE	NE	NE	NE	NE

Arrowhead up: upregulation; arrowhead down: downregulation; NE: not evaluated; *α*: plus an increase in VGLUT_2_-LI in small neuron profiles; *unpublished data; tissue has been processed and NPs quantified according to references [54, 60–62].

## Abbreviations

ATF-3: Activating transcription factor, type 3
CGRP: Calcitonin gene related peptide
CNS: Central nervous system
DA: Dopamine
DRG: Dorsal root ganglion
EAAT: Excitatory amin oacid transporter
EGFP: Enhanced green fluorescent protein
GC: Granular cells
IB4: Isolectin B4
IGLEs: Intraganglionic laminar endings
IR: Immunoreactive
KO: Knock-out
L: Lumbar
Li: Like-immunoreactivity
LSC: Lumbar sympathetic chain
LSN: Lumbar splanchnic nerve
MPG: Major pelvic ganglion
NaV 1.8: Voltage dependent sodium channel, type 1.8
NGF: Nerve growth factor
P_2_X_3_: P2X purinoceptor 3
PN: Pelvic nerve
PNS: Peripheral nervous system
SCG: Superior cervical ganglion
TH: Tyrosine hydroxylase
TL: Thoracolumbar
TRPA_1_: Transient receptor potential cation channel, subfamily A, member 1
TRPV_1_: Transient receptor potential cation channel, subfamily V, member 1
VGLUT: Vesicular glutamate transporter
Y1R: Neuropeptide tyrosine receptor, type 1
Y2R: Neuropeptide tyrosine receptor, type 2.
